# Development of an Ultra-Pure, Carrier-Free ^209^Po Solution Standard

**DOI:** 10.6028/jres.120.011

**Published:** 2015-07-23

**Authors:** R. Collé, R. P. Fitzgerald, L. Laureano-Perez

**Affiliations:** National Institute of Standards and Technology, Gaithersburg, MD 20899 USA

**Keywords:** alpha counting, lead-205, liquid scintillation (LS), measurements, polonium-209, radioactivity, standards

## Abstract

Ultra-pure, carrier-free ^209^Po solution standards have been prepared and standardized for their massic alpha-particle emission rate. The standards, which will be disseminated by the National Institute of Standards and Technology (NIST) as Standard Reference Material SRM 4326a, have a mean mass of (5.169 ± 0.003) g of a solution of polonium in nominal 2.0 mol▪L^−1^ HCl (having a solution density of (1.032 ± 0.002) g▪ mL^−1^ at 20 °C) that are contained in 5 mL, flame-sealed, borosilicate glass ampoules. They are certified to contain a ^209^Po massic alpha-particle emission rate of (39.01 ± 0.18) s^−1^▪g^−1^ as of a reference time of 1200 EST, 01 December 2013. This new standard series replaces SRM 4326 that was issued by NIST in 1994. The standardization was based on 4πα liquid scintillation (LS) spectrometry with two different LS counting systems and under wide variations in measurement and counting source conditions. The methodology for the standardization, with corrections for detection of the low-energy conversion electrons from the delayed 2 keV isomeric state in ^205^Pb and for the radiations accompanying the small 0.45 % electron-capture branch to ^209^Bi, involves a unique spectral analysis procedure that is specific for the case of ^209^Po decay. The entire measurement protocol is similar, but revised and improved from that used for SRM 4326. Spectroscopic impurity analyses revealed that no photon-emitting or alpha-emitting radionuclidic impurities were detected. The most common impurity associated with ^209^Po is ^208^Po and the activity ratio of ^208^Po/^209^Po was < 10^−7^.

## 1. Historical Introduction

The element polonium was the first naturally-occurring, radioactive element discovered. Marie Curie (nee Sklodowska) and Pierre Curie isolated the element in 1898 from pitchblende after separation of uranium and thorium [1]. Although the concept of isotopes was still unknown, they undoubtedly had observed ^210^Po from the ^238^U series. Forty-two known isotopes of polonium exist [2], and interestingly the longest-lived isotopes, ^208^Po and ^209^Po with half-lives of about 2.9 a and 125 a, respectively, were not discovered until 1947 to 1949 [3, 4], with the advent of light, charged particle (alpha and deuteron) induced reactions with accelerators.

Standards of these three important polonium isotopes have been and are important in many disciplines. At present, they are primarily of interest as calibration standards for alpha particle energy and alpha-emission rate measurements, and as low level tracers and separation yield monitors in radiochemical procedures for environmental measurements and geophysical studies. Early on, ^210^Po (138 d half-life) was one of the first widely used alpha sources in nuclear physics and radiochemistry research. Later both ^208^Po and ^209^Po were preferred because of their longer half-lives and since they could be used to trace ^210^Po for ^210^Pb assays.

The National Institute of Standards and Technology (NIST) — known between 1901 and 1988 as the National Bureau of Standards (NBS) — as the national standards laboratory and metrology institute of the USA, has been involved in radioactivity measurements since 1914 [5] and played a vital role in the development and dissemination of these polonium standards.

Collé et al. [6] in 1995 provided a brief historical overview of the NIST/NBS activities for ^210^Po and ^208^Po measurements, calibrations, and standards through the mid-1980s, as well the emerging need for a more desirable ^209^Po standard. For the past 25 years, various research and standards development work at NIST on ^209^Po has been extensive.

A two-year study of the long-term stability of carrier-free polonium solutions under various acidity conditions over periods of up to 9 years was reported on in 1993 by Collé [7]. That work was a precursor to the development of a carrier-free ^209^Po solution standard, which was disseminated by NIST as Standard Reference Material (SRM) 4326 [6, 8]. It is believed that this was the first standardization of ^209^Po by any national metrology institute and was the world’s first issue of a ^209^Po standard. The standardization was based on 4πα liquid scintillation (LS) spectrometry using a unique spectral analysis methodology that was specific for the case of ^209^Po [6]. The standardization work led to the identification by Collé et al. [9] of a delayed isomeric state in ^205^Pb, which has serious implications for LS counting of ^209^Po. At the same time, concern over aspects of the ^209^Po decay scheme led to investigations by Schima and Collé [10] on the branching ratios for alpha-particle decay to ^205^Pb and the electron capture (EC) decay to ^209^Bi, and the photonic emission probabilities per decay for the x-ray transitions accompanying the EC branch.

In 2005, measurements for the re-certification of SRM 4326 revealed a serious 25 % discrepancy in the then-known half-life of ^209^Po as established by two sets of precise, primary standardization measurements made approximately 12 years apart in the NIST laboratory [11]. Though the magnitude of the half-life error was surprising, it could be attributable to the fact that the only reported determination up until then was based on results that could not withstand critical scrutiny [11–13].

Around the same time period, NIST scientists began to work on the first primary standardization of ^210^Pb for SRM 4337 [14, 15]. One must appreciate that there is an intimate linkage between ^209^Po and ^210^Pb in application [11, 16]. Solution standards of ^209^Po are employed to trace the radiochemical yield of ^210^Po, the decay product of ^210^Pb, whose in-growth measurement is used for ^210^Pb assays. Standardization of ^210^Pb is particularly difficult and troublesome, so NIST scientists initiated an informal measurement comparison and bilateral exchange of ^210^Pb standards with the National Physical Laboratory (NPL) of the UK [16]. The exercise demonstrated good agreement, well within the respective measurement uncertainties.

In 2013, the issue of the half-life of ^209^Po was re-visited by a third primary standardization of the 1993 ^209^Po solution (SRM 4326). These further measurements confirmed the earlier report [11] on the error in the ^209^Po half-life. Coupled with the earlier standardization measurements, the results were used to derive a new half-life value of (125.2 ± 3.3) a [13]. This work was done in conjunction with the preparation of a new issue of ^209^Po (SRM 4326a) [17], which used a similar, but slightly revised and improved standardization methodology as reported on herein.

Table 1 gives a chronological summary of the various NIST/NBS studies and metrological activities involving the polonium isotopes, ^210^Po, ^208^Po, and ^209^Po, over the past 60 or more years.

This paper is intended to document and archive the details for the preparation and standardization of the new ^209^Po SRM 4326a series. The replacement of SRM 4326 was delayed for several years because of the unresolved issue of the ^209^Po half-life discrepancy and because of informal reports [21] from NPL of serious polonium solution instability problems using the same ^209^Po stock material.

## 2. Experimental Details

### 2.1 Experimental Design

**Fig. 1:**
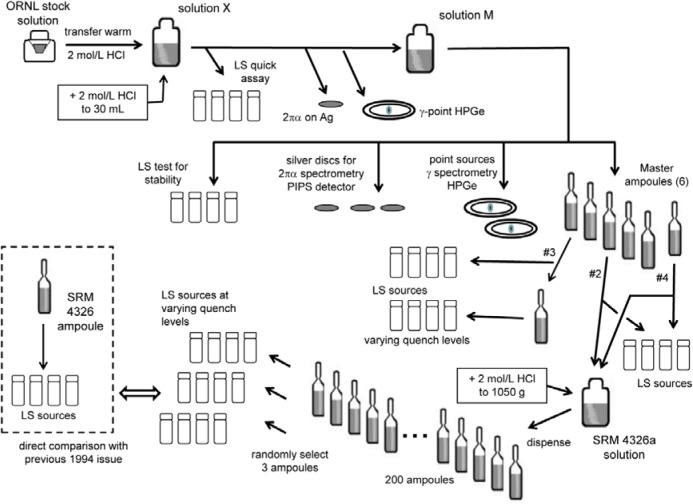
Experimental design and schema for preparation of the ^209^Po standards and counting sources used for the calibration.

Figure 1 illustrates the experimental design and scheme for the preparation of SRM 4326a and the various counting sources used for the standardization. The plan was largely designed to test that the ^209^Po solution would be stable and invariant with dilutions, sampling, and temporal dependence. This was in part driven by concerns raised by NPL over the stability of the same ^209^Po stock material that was reported to be unstable on dilution with HNO_3_, with 5 mol▪L^−1^ HCl and with or without Te carrier ions, and with 8 mol▪L^−1^ HCl, speculating that the ^209^Po “was in the form of colloid or polymer, or perhaps contained a chemical impurity that was affecting the stability” [21].

It was concluded that we would proceed with using 2 mol▪L^−1^ HCl solutions as was shown to be stable by Collé [6] and as used for SRM 4326 in 1993 [5]. Nevertheless, to build in safeguards, aliquots were taken at various stages along the way and at various times to prepare LS counting sources: e.g., on first diluting the stock material to make solution X; at the time of filling the master ampoules from solution M; sampling the master ampoules when used to prepare the SRM 4326a solution by dilution; and from three of the 4326a ampoules. This is illustrated in Fig. 1. All of these LS counting sources were gravimetrically linked to each other and to solution M that was contained in the master ampoules. Additional point sources were prepared for α-particle counting and spectrometry and for photonic emission spectrometry.

### 2.2 Preparation of SRM 4326a

The ^209^Po stock material was obtained from Oak Ridge National Laboratory. It consisted of nominal 5 mol▪L^−1^ HNO_3_ in a small volume (≈ 0.8 mL) that was transferred and diluted to approximately 30 mL with warmed (≈ 50 °C) 2 mol▪L^−1^ HCl. The solution X and solution M (see Fig. 1) referred to an identical solution contained in the same 50 mL bottle, but was given separate designations to represent that it was sampled at different times.

All of solution M was put up into six flame-sealed master ampoules, two of which were used to prepare SRM 4326a, another was partly used for additional sampling, and its remainder and the other three were stored for future ^209^Po SRM issues.

The dispensing solution for SRM 4326a was prepared by a careful gravimetric dilution using the two master ampoules and a 2.0 mol▪L^−1^ HCl diluent. The HCl solution used for the dilution was prepared from high-purity “TraceMetal™ Grade” concentrated hydrochloric acid (Fischer Scientific, Hampton, NH)^1^ and low-conductivity, deionized-distilled water. The HCl solution density (1.032 ± 0.002) g▪ mL^−1^ at 20 °C) was determined from fitting the slope of measured cumulative masses obtained with a series of dispensed volumes from a class A 50 mL buret.

**Fig. 2:**
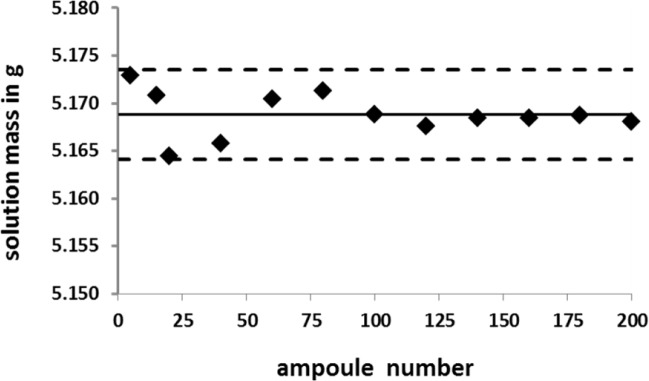
Contained solution masses of the ^209^Po standards as a function of ampoule filling as dispensed with a single-syringe automatic dispenser. The solid line is the mean of 12 determinations, spaced approximately 20 ampoules apart, across the 200 filled ampoules. The broken lines are upper and lower limits for plus and minus two standard deviations of the *n* = 12 distribution. The greater dispersion in ampoules numbered < 100 is attributed to minute (< 5 mm^3^) air bubbles initially occluded within the syringe.

The SRM 4326a solution was dispensed into approximately 200 borosilicate glass ampoules using a Hamilton Microlab 900 single-syringe automatic dispenser (Hamilton Bonaduz A.G., Bonaduz, CH). Twelve, or about one out of every 20^th^ ampoule that was filled, had been pre-weighed and then post-weighed to obtain an estimate of the dispensing precision and contained solution mass. Weighing was performed with an electronic analytical balance. Figure 2 provides the mass results as a function of filling order. The mean mass^2^ was (5.169 ± 0.003) g, where the uncertainty given here is two times the standard deviation for the *n* = 12 sample distribution. After filling and weighing, the ampoules were flame sealed, inspected, autoclaved, and labelled.

#### 2.2.1 Dilution Factor Determination

**Fig. 3:**
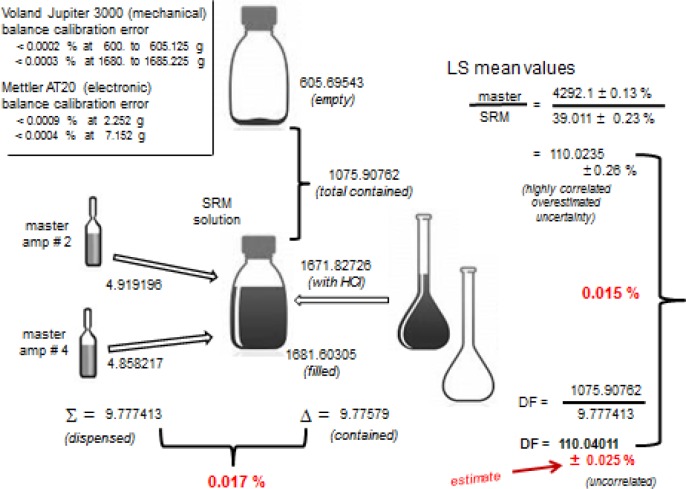
Determination of the gravimetric dilution factor for preparing the SRM 4326a dispensing solution from solution M master ampoules #2 and #4. See Fig. 1 and text (Sec. 2.2.1) for details.

The determination of the gravimetric dilution factor in preparing the SRM 4326a dispensing solution from solution M is illustrated in Fig. 3. It uses an approach that considers the measurement of both contained and dispensed masses. The level of detail included here is intended to document the typical procedures used by this laboratory to carefully perform a larger-volume dilution, such as needed for the preparation of a dispensing solution that is used to make a series of standards. It illustrates the realistic assessment of the uncertainty on such a procedure.

An electronic microbalance (Mettler AT20) was used to weigh the dispensed master solution (from the two 5 mL master ampoules) and a large capacity (3 kg) mechanical balance (Voland Jupiter 3000) was used to obtain the contained mass from the two ampoules and the total solution mass. All appropriate air buoyancy corrections were applied for the masses that are shown at the various steps in Fig. 3. The master solution dispensed mass from the ampoules was obtained from mass differences with an aspirating polyethylene pycnometer. The weighing with the large capacity balance for each mass was based on the average of three readings. At the time of the measurements, both balances were checked for possible calibration errors with OIML Class E1 standard weights in the exact mass ranges used for the determinations (single substitution with sensitivity weights). The large capacity balance was evaluated in the mass range of the empty bottle (605 g) and the mass range for the filled bottle (1681 g). Maximum deviation relative bias errors of < 0.0002 % and < 0.0003 %, respectively, were found. The microbalance was evaluated at the mass ranges for empty and filled pycnometer, with errors of less than < 0.0009 % and < 0.0004 %, respectively. The mass data from the weighings yield a dilution factor *DF*:

$$DF=\frac{1075.90762g}{\left(4.919196+4.858217\right)g}=110.0401.$$

The relative difference in the dispensed and contained mass of the master solution (nominal 9.78 g) was found to be 0.017 %. As noted in Fig. 3, the gravimetric dilution factor was subsequently verified by LS measurements of aliquots of the two solutions to within 0.015 % (Sec. 4.2). The estimated relative standard uncertainty on the dilution factor was taken to be 0.025 %, in part based on the long-term experience of the metrologist for the replicate measurement repeatability and the zero-tare stability of the two balances (though confirmed by the present observations). The uncertainty on air buoyancy was negligible as was most of the other balance and environmental effects (non-linearity, sensitivity tolerance, temperature coefficient, etc.), and with this approach there was no need to speculate on unquantifiable other effects, e.g., evaporation losses, electrostatic influence, or eccentricity, etc.

### 2.3 Counting Sources and Instruments

#### 2.3.1 Liquid Scintillation Spectrometry

The LS counting sources were obtained from aliquots of solution X, solution M, from three of the master ampoules, and from three randomly selected SRM 4326a ampoules, all at differing times. In all, 13 different series of sources were prepared as noted in Table 2. Each series consisted of three sources, with the exception of series M3 that had six quench-varied sources. The source cocktails were gravimetrically prepared with aliquants of the ^209^Po solutions as dispensed by pycnometer and measured with a electronic microbalance (Mettler AT20). Other cocktail components were weighed with a mechanical analytical balance (Mettler B5). The cocktails, contained within 20 mL glass LS vials, were prepared with commercially available scintillation fluids, either “Ready Safe” (Beckman/Perkin-Elmer, Waltham, MA) designated as RS or “UltimaGold AB” (Perkin-Elmer) designated as UG. Typical source composition consisted of 10 mL of either RS or UG fluid, a ^209^Po solution aliquant of 0.03 g to 0.06 g for the higher level series (X, L, M’s) and 0.2 g to 1.1 g for the SRM-level solutions, along with additions of 2 mol▪L^−1^ HCl to maintain aqueous fractions in the range 5 % to 10 %. In some cases, chemical quenching was varied within a set of sources or between two matched sets by addition of varying amounts of a nominal 30 % ethanolic solution of nitromethane as an imposed quench agent. Variation in the acidity between series S89a and S89b was also adjusted by additional water. The cocktail compositions for the various series are summarized in Table 2, along with the origin of the source material for the series and the preparation date.

Each source was measured for typically three counter cycles (replicates) on at least one measurement occasion on each of two different LS spectrometers. They were:

Beckman LS 6500 (Beckman Coulter, Fullerton, CA), designated as counter “B”;
Wallac 1414 Winspectral (Perkin-Elmer, Wesley, MA), designated as counter “W”.

More than one counter is typically used in our laboratory for any given measurement to hopefully demonstrate that results are independent of the operating characteristics of the spectrometer (detection threshold, photomultiplier efficiency, deadtime, amplification, signal conversion, etc.) which vary substantially between instruments. Laureano-Perez et al. [14] have described the differences in the characteristics of these instruments.

To evaluate temporal dependence, independent of series variability and instrument, series X, L, M2, and M4 were measured on two separate measurement occasions on counter B.

#### 2.3.2 Photonic Emission Spectrometry

Point sources for impurity analyses by high-purity intrinsic germanium (HPGe) spectrometry were prepared at two places in the scheme (see Fig. 1). The sources were prepared by depositing 35 mg to 45 mg of the solutions onto annular source mounts backed with 0.06 mm thick (≈ 7 mg▪cm^−2^) plastic tape (glued polyester tape). After air drying the deposit, the sources were covered and sealed with an identical layer of the tape. The diameter of deposits was typically < 0.5 cm. One of the master ampoules was also directly measured with a spectrometer for a 7 day interval to set impurity limits.

Measurements were performed with the NIST HPGe detector “B” at 6 cm for the point sources, and with detector “X” with side-mount for the ampoule. The detectors and spectrometry procedures used by NIST have been described in detail by Pibida et al. [24, 25].

#### 2.3.3 Alpha-particle Counting and Spectrometry

Silver foils mounted on 2.5 cm diameter stainless steel (SS-306) disks were used as the matrix for α-counting and α-spectrometry. Solution aliquots were deposited and air dried, forming solid sources by spontaneous electrochemical deposition with irregular deposits of breadths up to 1.5 cm, covered with VYNS films with a surface density of about (10 to 20) µg▪cm^−2^. These sources were prepared at the same time as those for HPGe spectrometry (see Fig. 1).

Some additional mixed sources of ^209^Po, ^208^Po and ^241^Am were prepared on silver and other matrices at later stages of this work, and will be discussed in Sec. 4.3.2.

Alpha counting was performed with a 2π multi-wire proportional counter filled with P-10 counting gas (90 % argon, 10 % methane) to 0.1 MPa (1 atm). The spectrometry was done with 450 mm^2^ passivated ion-implanted planar Si detectors (PIPS) at fixed geometry distances.

## 3. Standardization Methodology

All of the measurement results for the standardization are given with respect to the massic concentration of solution M. Every counting source was linked to M through mass determinations and the gravimetric dilution factor (Sec. 2.2.1), as illustrated within Fig. 1.

The procedure used to determine the massic alpha-particle emission rate for the ^209^Po solution from the LS data required spectrometry with spectral interpretation and analyses rather than just LS counting. The procedure largely follows that used by Collé et al. [6] for the standardization of SRM 4326 in 1994. Previously, spectra were analyzed by dividing each spectrum into two regions with a cut-off just above the response due to the electrons from the ^205^Pb 2 keV transition and the electronic noise. Typical spectra and analysis details are given in full in Ref. [6]. A reproducible and constant massic rate, attributed to the alpha emission, was obtained for each measurement trial on subtracting this lower energy region from the total spectrum. This rate was then corrected for the response due to radiations from the 0.45 % electron-capture branch using a correction factor, taken as *k* = 0.9988 with an estimated uncertainty of about 0.05 %. The derivation of this *k* correction is given in an Appendix in Collé et al. [6].

For the present work, the earlier 1994 procedure was modified to make it a more robust spectral analysis procedure, which in turn resulted in an order of magnitude smaller *k* correction. It is significant to appreciate that the standardization measurements for this present work were made concurrently with the 2013 re-standardization of SRM 4326 that was used for the recent ^209^Po half-life determination [13]. Analyses of that data with either method gave results that were invariant and statistically equivalent to within their respective precision estimators.

The spectral analyses procedure developed for the LS-based standardization of ^209^Po is unique and specific for the case of ^209^Po, as driven by the characteristics of the ^209^Po decay scheme.

### 3.1 ^209^Po Decay Scheme

**Fig. 4:**
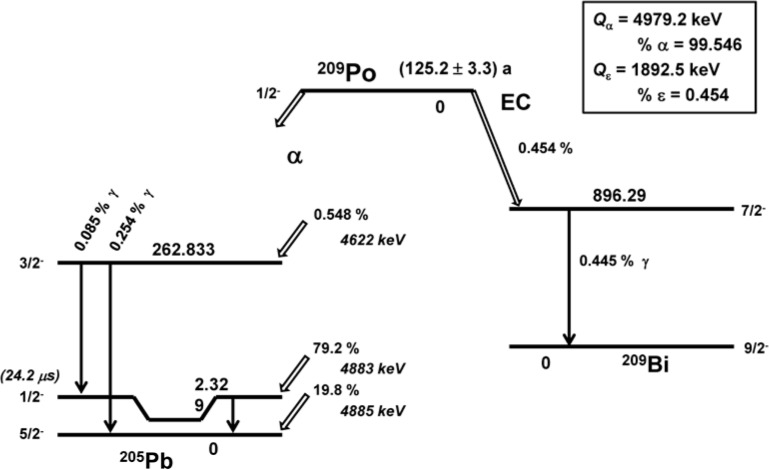
^209^Po decay scheme showing the alpha decay branch to ^205^Pb and the electron capture (EC) to ^209^Bi, as adapted from data in Ref. [26]. The ^209^Po half-life is that from Ref. [13]. A summary of the ^209^Po radiations from both branches is given in Table 3.

Figure 4, adopted from data in Ref. [26], shows the decay scheme of ^209^Po. The LS-based standardization of ^209^Po must adequately account for the 2.3 keV delayed isomeric state (24.2 µs) in ^205^Pb and for the radiations accompanying the 0.454 % EC branch to ^209^Bi. Table 3 summarizes all detectable radiations arising from the decay of ^209^Po. The design of the spectral analysis method as well as the EC branch correction (see Appendix) were based on an evaluation of these radiations.

### 3.2 Spectral Analyses

**Fig. 5:**
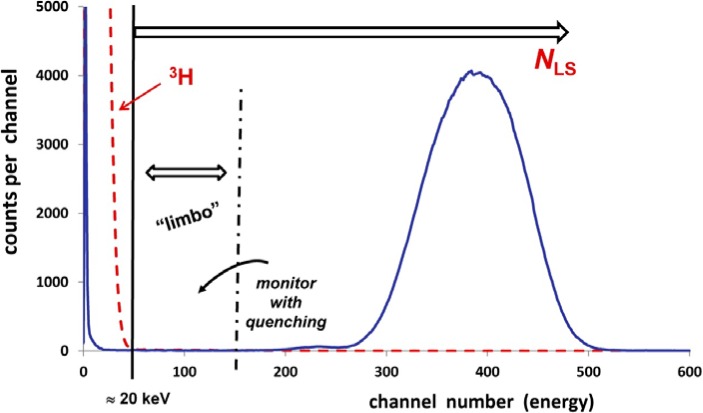
Three regions in the LS spectrum of ^209^Po as used for the spectral analysis. The first region is defined as that below about 20 keV, the beta endpoint energy of ^3^H, as obtained by spiking the ^209^Po LS cocktail with a tritiated water source. The upper region above the ^3^H cut-off covers the LS response *N*_LS_ attributed principally to alpha particles and includes the “anomalous bumps” [6] on the low energy shoulder. A higher-energy window set just below the apparent alpha peak is used to define a “limbo” region. On imposing additional chemical quenching on the ^209^Po spectrum, the response in the window labelled “limbo” is monitored to validate that there is no substantive increase in count rate in the region below 20 keV. Refer to text (Sec. 3.2).

For the spectral analyses, the ^209^Po spectrum from each series was divided into three regions, as shown Fig. 5. The counting windows for these regions were individually set for each series and for the LS spectrometer that was used.

A window was set at the uppermost region to include the major portion of the LS response due to the alpha emission, which encompassed the evident and predominant alpha peak. This region includes any “anomalous bumps” on the low energy alpha peak shoulders, which were shown by Collé et al. [6] to be attributable to alpha interactions. The window setting was evaluated by adding an imposed chemical quenching agent to the ^209^Po cocktail and ensuring that there was no significant count rate loss in this upper region or any increase in the adjacent middle region.

The lowermost region was established by spiking a ^209^Po cocktail with an aliquant of a tritiated water standard [27]. The observed ^3^H beta spectrum endpoint at about 20 keV was used to set the upper edge of this lower region. With this, one could be assured that all electrons arising from ^209^Po decay below this threshold were not detected. This eliminated much of the uncertainty in correcting for the LS response due to radiations from the EC branch. Refer to the Appendix (Sec. 6).

The middle region was termed “limbo” (without deference to calling it “the oblivion”) to signify that it is an intermediate state or an uncertain region that is used to await a decision. This region was initially used to set the upper window of the alpha region with changes in quenching. Once the windows were set for any given series, count rate changes in this window region were monitored for any spillage from the upper alpha region into limbo. It can be anticipated that the contribution of any alpha emission into the region below the ^3^H cut-off would be less than that seen in the limbo region.

The integral counts *N*_LS_ above the ^3^H cut-off (i.e., the sum of both upper windows) in the spectrum for any counting source was used to derive a massic LS rate *R*_LS_ given by

$$R_{\text{LS}}\frac{N_{\text{LS}}-N_{B}}{m▪t_{c}▪\exp\left[-\lambda \left(\Delta t\right)\right]},$$ (1)

where

*N_B_* is the integral counts in a background spectrum for a matched blank obtained with the identical window settings used to obtain *N*_LS_;
*m* is the aliquant mass of the ^209^Po solution used to prepare the LS counting source;
*t*_c_ is the counting livetime for the spectrum accumulation;
*λ* is the ^209^Po decay constant given by ln(2)/*T*_1/2_, with *T*_1/2_ = (125.2 ± 3.3) a [13]; and
*∆t* is the time interval from the counting midpoint time to the reference time (1200 EST, 1 December 2013).

The background-subtracted and decay-corrected massic LS rate *R*_LS_ could be related to the massic alpha emission rate through the application of a very small correction factor k given in the Appendix:

$$E_{\alpha }=kR_{\text{LS}}.$$ (2)

For least or minimally quenched samples, the relative count rates for an entire spectrum in the three regions typically were: about 96 % to 98 % for the upper alpha window; 2 % to 4 % for the lowest, ^3^H-cut-off window; and less than a few tenths of a percent (typically 0.1 %) in limbo.

## 4. Results and Discussion

### 4.1 Liquid Scintillation Spectra

**Fig. 6:**
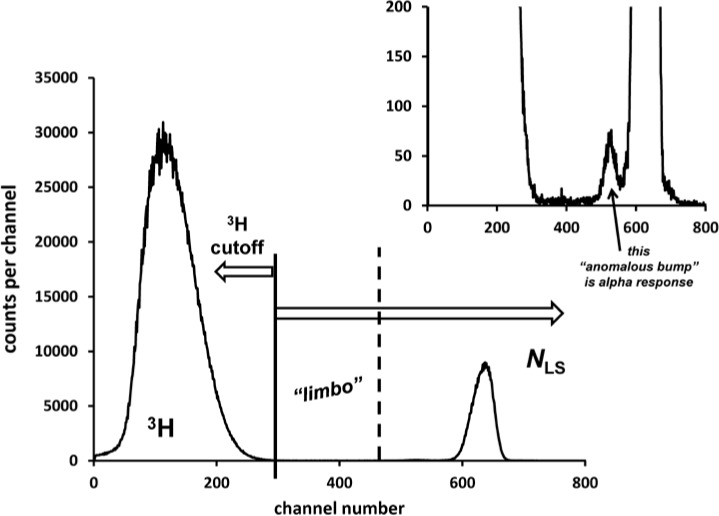
Typical spectrum obtained with the Wallac LS spectrometer as used to set the windows for the three regions for series M2. The channel numbers correspond to a logarithmic energy scale, of unequal energy bins between channels. The ^209^Po cocktail was spiked with ^3^H to establish the lower 22 keV cut-off. The inset shows an example of an “anomalous bump” [6] which is part of the alpha response.

**Fig. 7:**
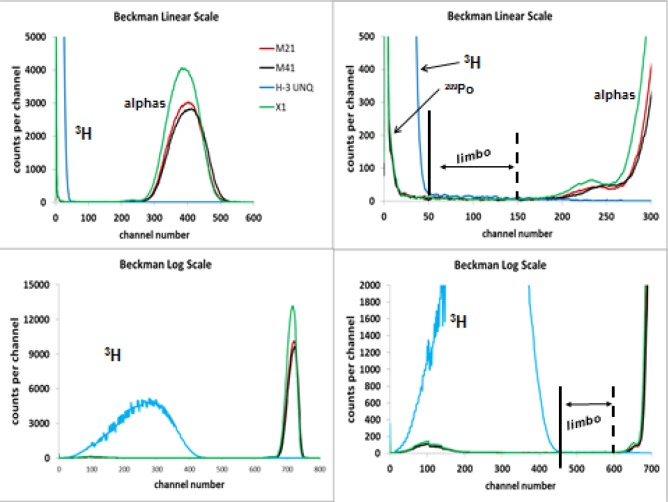
LS spectra of ^209^Po and ^3^H as obtained with the Beckman spectrometer for sources from several series (X, M2, M4) shown in both linear and logarithmic energy scales. The clear definition of regions is apparent.

Typical ^209^Po LS spectra obtained with the Wallac (W) and Beckman (B) spectrometers are given in Figs. 6 and 7. Both instruments record the spectral data (counts per channel vs. channel number) in logarithmic form through sum-coincident pulse amplification and analog-to-digital conversion, though both have the capability of converting the spectra into a linear energy scale on the channel axis. The relative magnitude of peaks may deceive on first appearance. In a logarithmic spectrum, the abscissa is a logarithmic energy scale with each channel having a different energy bin width. In a linear spectrum, the energy bin widths are the same magnitude.

Figure 6 is the logarithmic spectrum obtained with the Wallac (W) spectrometer for a source from the M2 series that was spiked with ^3^H. The three spectral regions are clearly delineated.

Figure 7 illustrates typical spectra from the Beckman (B) spectrometer in both linear and logarithmic displays. The sources for the three ^209^Po spectra were unspiked and obtained from series X, M2, and M4. A separate spectrum of a ^3^H source is also shown. Comparison of the upper and lower left-hand side spectra for the relative sizes of the peaks for the ^209^Po alphas and the ^3^H beta spectrum clearly shows the scaling effect.

**Fig. 8:**
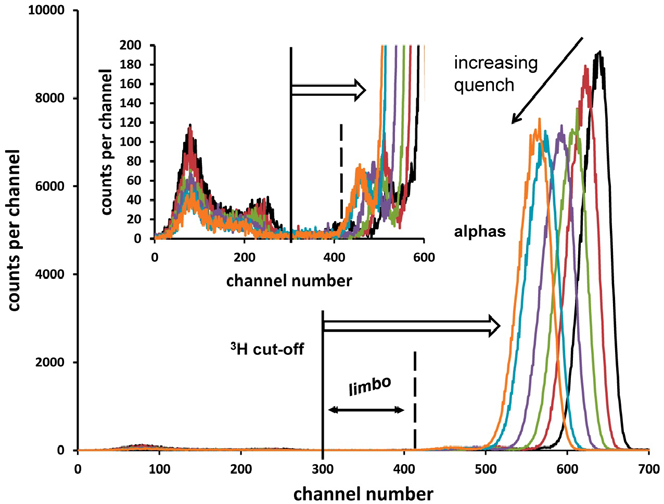
LS spectra with Wallac spectrometer for series M3 showing the effect of imposed chemical quenching. The windows were set with a ^3^H-spiked, highly quenched counting source. The inset shows the “anomalous bumps” [6] on the low energy shoulders of the alpha peaks, and the response below the ^3^H cut-off from the 2.3 keV transition and the EC branch radiations.

The effect of imposed chemical quenching on the ^209^Po LS spectrum is illustrated in Fig. 8. These spectra from the Wallac (W) spectrometer were obtained from the six quench varied sources in the M3 series. The limbo region, even under extreme quenching, is clearly defined. The windows had been set using a similarly prepared ^209^Po source of largest quench that had been ^3^H-spiked.

### 4.2 Massic Alpha-Particle Emission Rate

**Fig. 9:**
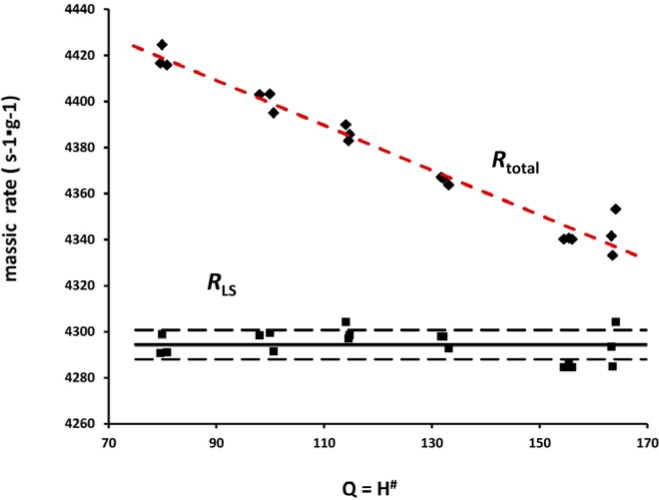
LS results for the series M3 as measured with the Beckman spectrometer, showing the effect of imposed chemical quenching on the massic rate for the total spectrum *R*_total_ and the constant massic alpha rate *R*_LS_. The dashed red line for *R*_total_ is only meant to guide the eye. The solid and broken lines for *R*_LS_ correspond to the mean and the upper and lower limits for ± 2 standard deviations. The rates are plotted against a quench indicating parameter *Q*, given as the Horrocks number *H^#^*.

The results of spectral analyses for series M3 sources with the Beckman (B) spectrometer, as an example of typical data, are shown in Fig. 9. Although the massic rate for a total spectrum varies considerably with quenching, the extracted massic alpha rate is constant.

The sources from 13 solutions, sampled at various times and locations in the scheme (Fig. 1), were counted on both spectrometers with a few repeats to yield 31 mean values of the decay-corrected massic α emission rate *E*_α_ at a reference time of 1200 EST, 01 December 2013. The means were grouped by the series, as measured with a given spectrometer (B or W), and at midpoint measurement time *T*. Table 4 provides the results for these 31 mean determinations. Each mean was derived by first averaging the three (typical) replicate measurements (i.e., number of counter cycles) for each source in a series, and then averaging across the three sources. The precision estimator for each mean value in Table 4 was derived by computing the standard deviation of the mean (sdm) for the replicate measurements for a given source, taking a typical sdm value (defined to be the average of the mean sdm and median sdm), and adding it in quadrature to the between-source standard deviation computed from the three (or 6) source mean values.

All of the *E*_α_ results of Table 4 are given in terms of the massic concentration for solution M. The two levels of solution — those for M (series X, L, M2, M4, M3, M3a, M3b) and those for the SRM (series S17a, S17b, S89, S178, S89a, S89b) — were linked by the well determined dilution factor (Sec. 2.2.1). The mean for the *n* = 19 M values is 4292.1 s^−1^▪g^−1^ with a standard deviation of the mean of ± 0.13 %, while that for the *n* = 12 SRM values is 39.011 s^−1^▪g^−1^ ± 0.23 %. This gives a LS-based M/SRM ratio of 110.0235 ± 0.26 %, which agrees with the gravimetric dilution factor to within 0.015 %. The M/SRM LS-based ratio is highly correlated and the uncertainty cited here for it is an overestimate. See Fig. 3.

In addition to the deliberately imposed chemical quenching to test the independence of *E*_α_ on quench level, it should be noted that the two activity levels of solutions have slightly different acidities that also affect quenching. The M-level solutions (series X, L, and M’s) contain about 0.1 mol▪L^−1^ HNO_3_ in 2.0 mol▪L^−1^ HCl upon transfer and dilution of the original HNO_3_-based Oak Ridge stock material. The SRM-level solutions on further dilution with HCl reduced this HNO_3_ content to approximately 0.001 mol▪L^−1^.

**Fig. 10:**
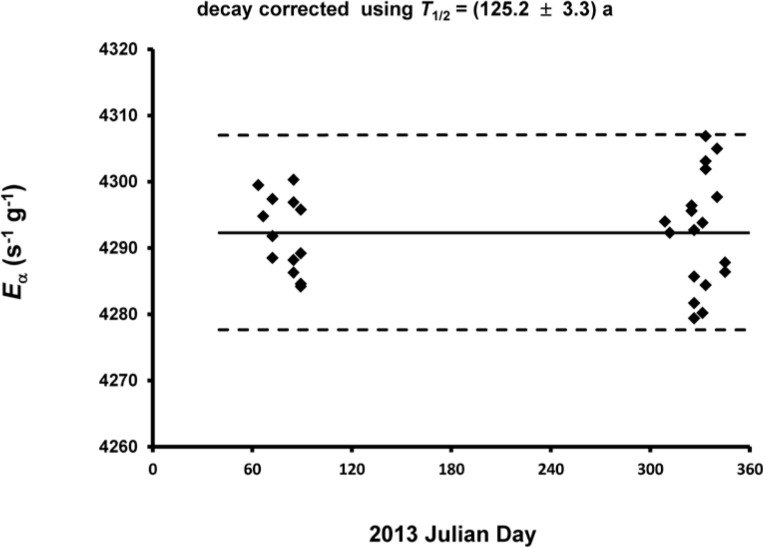
The *n* = 31 determinations of decay-corrected massic alpha emission rate *E*_α_ of solution M at reference time 1200 EST, 01 December 2013 as a function of the measurement midpoint time *T*, in 2013 Julian Day. The solid and broken lines correspond to the mean and the upper and lower limits for ± 2 standard deviations.

**Fig. 11:**
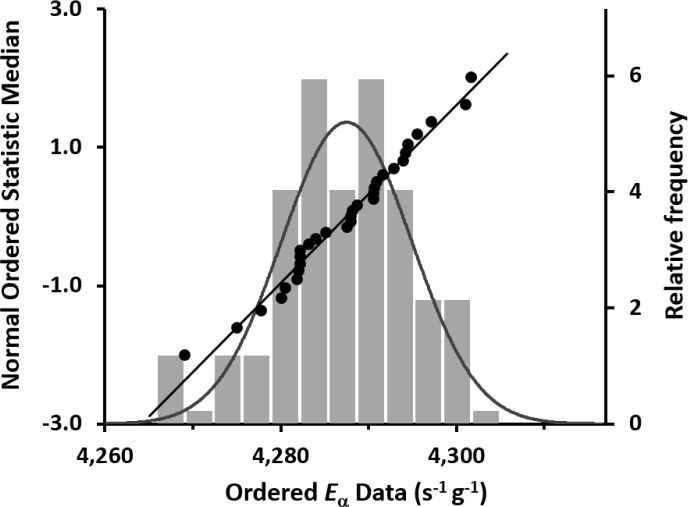
The 31 mean values of the massic alpha emission rate *E*_α_ for the master solution M used to prepare ^209^Po solution standard SRM 4326a, as represented in a three-part Fitzgerald plot (see Ref. [13]) that overlays the probability plot correlation coefficient (PPCC) graph and a frequency histogram of the same data. A normal distribution *N* (4292.3, 7.3) is superimposed on the histogram.

As given in Table 4, the grand mean for the *n* = 31 determinations is *E*_α_ = 4292.3 s^−1^▪g^−1^ at the 1200 EST, 01 December 2013 reference time. The overall effect on *E*_α_ of including the response in the limbo region in *N* across all *n* = 31 determinations was to increase *E*_α_ by 0.113 %. A plot of the decay-corrected *E*_α_ values as a function of measurement midpoint time *T* is given in Fig. 10. The typical precision estimator (as a standard deviation of the mean) for the within-determination variability is 0.14 %. The between-determination standard deviation for the *n* = 31 mean values is 0.17 % (equivalent to a standard deviation of the mean for the grand mean). Figure 11, in the form of a Fitzgerald plot [13], shows the normal probability test for the results based on the probability plot correlation coefficient (PPCC) [28]. The data passes both an Anderson-Darling goodness of fit test and a Wilk-Shapiro test for normality (p ≈ 0.76) at 95 % confidence level [29].

A curious aspect of the original data is that although the measurements cover only a period of 276 days, they are sufficient to crudely estimate the ^209^Po half-life. A fit of the *E*_α_ versus *T* (before decay corrections) yields a half-life of (128 ± 21) a, which fortuitously only differs from the definitive half-life determination of Collé et al. [13] by about 2 %. Such a determination of a half-life over a time period that is only a very small fraction of one half-life often provides a fitted result close to a “true” central value due to the mathematical nature of distributions and stochastics. Such a determination, however, is rarely meaningful or robust since it is impossible to adequately assess possible long-term influences for a complete and rigorous uncertainty assessment [30].

### 4.3 ^209^Po Solution Stability

As stated previously, a large aspect of this work involved testing and ensuring that the carrier-free polonium solution was stable. Figure 1 highlighted the complexity of the designed scheme in terms of sampling and preparing LS sources at various times and under other conditions, preceding and following solution dilutions and solution transfers. Figure 10 shows the invariance with time, in two groupings separated by nearly 220 days. The first grouping of results (at times in March, 2013) was only from solution M series, while the second group (in November to December, 2013) was primarily from the gravimetrically linked lower-level SRM series. A linear fit of *E*_α_ vs *T* has a slope of 0.0012 s^−1^▪ g^−1^/day and a squared Pearson correlation coefficient of *r*^2^ = 0.0004. From this, one can conclude that solution stability is demonstrated by Fig. 10 because of the time independence, as well as on dilution and repetitive sampling.

**Fig. 12:**
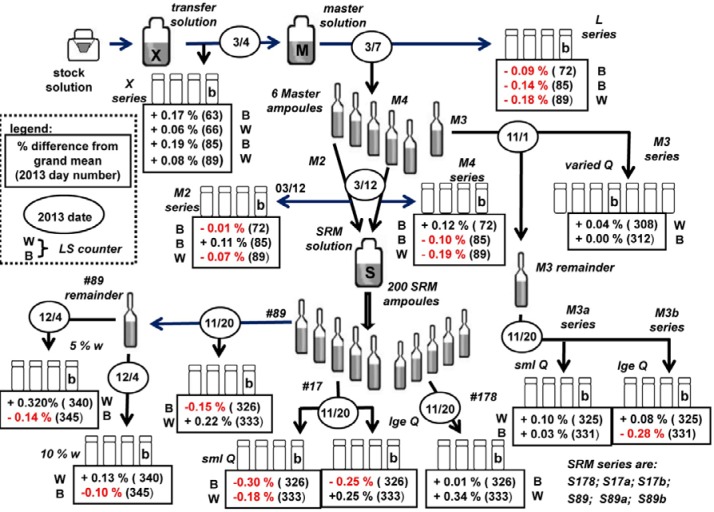
This extremely detailed figure illustrates the complexity of the sampling scheme used to evaluate and ensure ^209^Po solution stability. Each of the 31 mean determinations (Table 4) of the massic alpha emission rate *E*_α_ is shown in terms of % differences from the grand mean and as a function of source series, including quench conditions; 2013 sampling date; LS counter used for the measurements (B or W).

This is evidenced even more convincingly in Fig. 12, which summarizes the *n* = 31 mean values of *E*_α_ in terms of its location in the Fig. 1 scheme. It provides the *E*_α_ differences in (± %) from the grand mean of *E*_α_ = 4292.3 s^−1^▪ g^−1^ for each series, specifying the sampling date, the quench condition for the LS cocktails, and the LS counter (B or W) used for those measurements. Close examination reveals no significant differences in any of the 13 series, or with sampling time, or with quench condition of the counting sources or with the counter used. Various subset means and variances — grouped by solution sampled, scintillation fluid used (RS or UG), high and low quench conditions, spectrometer used (B or W), and measurement time — were tested for differences in *E*_α_ by *t*-and *F*-tests and by sequential one-way analysis of variance (ANOVA) techniques. None of the test results indicated that there were any statistically significant differences in any of the tested sub-grouped means and variances, with the exception of a slight systematic difference between the initial X and L series. Table 5 shows the invariance in *E*_α_ for various unaggregated subgroups for the principal measurement variables.

It is apparent that carrier-free polonium in 2 mol▪L^−1^ HCl constitutes a stable solution, as originally described by Collé [7].

### 4.4 Impurity Analyses and Confirmatory Attempts

#### 4.4.1 Photonic Emission

Photonic emission spectrometry using HPGe detectors (Sec. 2.3.2) was primarily intended for radionuclidic impurity analyses to ensure the ultra-purity of the ^209^Po solutions used for the SRM.

The only photons from ^209^Po decay are: the 260.5 keV (0.254 %) and 262.8 keV (0.085 %) γ rays in ^205^Pb; the 868.28 keV (0.445 %) γ ray in ^209^Bi; and the < 88 keV Pb and Bi x rays (Table 3). These were the only photons detected with the point source with the B detector in a 42-hour count or with the M2 ampoule on a side-mount with the X detector in a 6.8-day count. No other photons were detected. The estimated lower limits of detection in the energy region 30 keV ≤ Eγ ≤ 2000 keV ranged from (0.001 to 0.002) s^−1^▪ g^−1^, except in the region 880 keV ≤ Eγ ≤ 910 keV where the limit was 0.003 s^−1^▪ g^−1^. Therefore, on inclusion of the solution M to SRM dilution factor, the ratio of detection limit to ^209^Po activity in SRM 4326a in all energy regions 30 keV ≤ Eγ ≤ 2000 keV is 0.003 % to 0.008 %.

For improvement in detection sensitivity for the impurity analysis, the sources were not counted in the standard geometries. As a result, the ^209^Po massic activity derived from measurements of the three γ rays had a relatively large standard uncertainty of ± 8 %. The result was within −1.5 % of ^209^Po massic activity for SRM 4326a (corrected for the 99.548 % α branching). This was intended to confirm the massic alpha emission rate obtained from the LS spectrometry, but the large uncertainty on the γ-ray spectrometry precludes much of a conclusion.

#### 4.4.2 Alpha-Particle Emission

The four 2πα point sources on Ag (one from solution X and three from M, see Fig. 1) had serious surface irregularities and apparent non-uniform solid deposits, attributable to unknown chemical impurities in the solutions or chemical reactions between the Ag matrix and deposited HCl solutions. As a result, multi-wire proportional counting gave seriously unsatisfactory results. The range in the α massic emission rate for the four sources with two replicates was 2.4 % and the average from the LS-based *E*_α_ differed by −4.2 %. Although the sources had been covered with VYNS films, it is possible that there was some unaccounted loss of Po by volatility during or after preparation.

A second attempt at confirming the LS-based *E*_α_ by proportional counting was attempted with the use of more carefully prepared sources. In this case, the ^209^Po sources were prepared in conjunction with ones prepared from a ^241^Am solution standard [31]. The sources were made as ^209^Po alone, as ^241^Am alone, and as a mixed source of ^209^Po + ^241^Am. These consisted of the same 2.5 cm SS-306 disks, coated with a thin sputtered Pt layer. To achieve more source deposit uniformity, the solutions were deposited with both a Ludox (colloidal silica) seeding agent and Polysorbate 20 (a surfactant). On normalization to the known ^241^Am α emission rate, the ^209^Po α emission rate from proportional counting on the two sources (^209^Po alone and the mixed ^209^Po + ^241^ Am) agreed with the LS-based *E*_α_ to +0.2 % and +0.7 %, respectively.

Unfortunately, these Pt sources had surface irregularities even worse than those on Ag, and were still unsuitable for high resolution α spectrometry. The problem this time was attributed to the fact that the very thin Pt coating was sufficiently porous for the acidity content of the ^209^Po and ^241^Am solutions to attack the underlying SS disks. Compounding this was the complication that the ^209^Po solution at 2.0 mol•L^−1^ HCl and the ^241^Am solution at 0.9 mol•L^−1^ HNO_3_ formed a dilute, highly corrosive aqua regia. A further trial was attempted by covering the Pt-coated disks with 20 µg▪cm^−2^ VYNS before depositing the solutions. This time for the mixed ^209^Po + ^241^Am sources the ^241^Am solution was deposited first and dried before the addition of the ^209^Po solution. Many other source preparation attempts were tried, including use of plastics and even glass matrix backings, all without success. These trials also included attempts at high resolution α spectrometry confirmations of the LS-based *E*_α_ by comparisons with known solutions of both ^241^Am and ^208^Po. Future work will undoubtedly require preparation of electrodeposited sources.

**Fig. 13:**
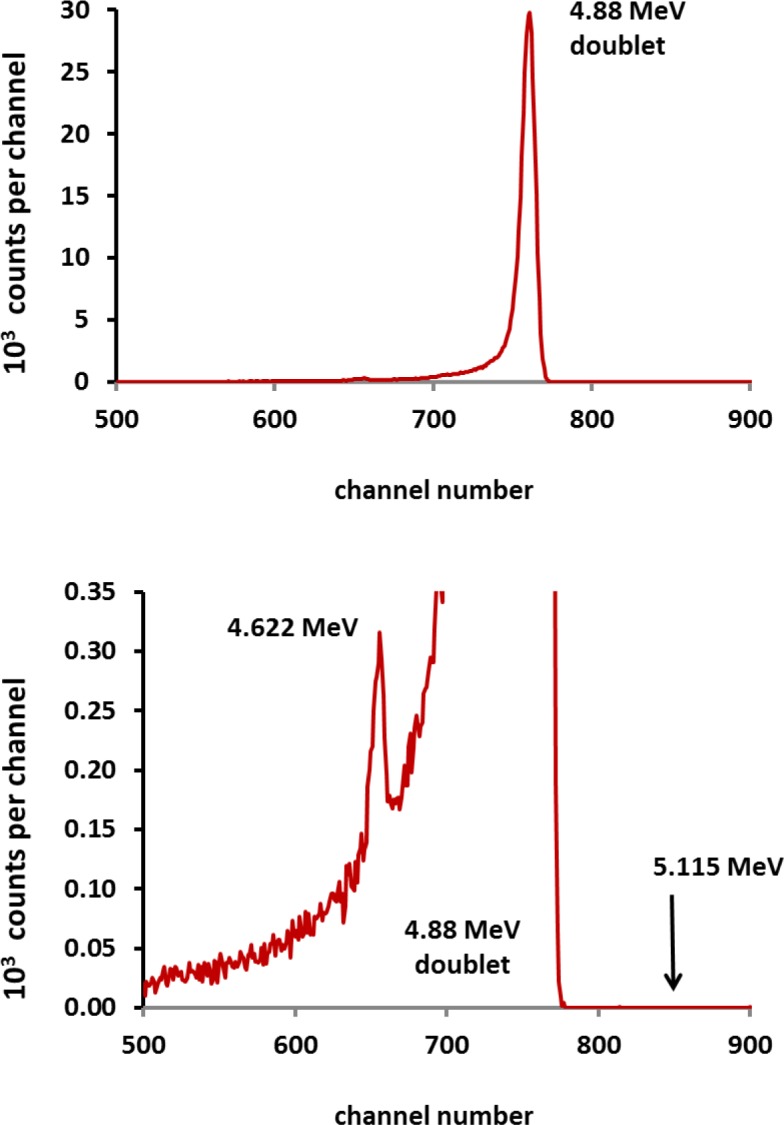
Typical high-resolution α spectrum obtained with Ag-backed point source of solution M on a PIPS detector. The spectrum could be used to set an impurity limit for the ^208^Po α transition at 5.112 MeV. Despite the serious low-energy tailing on the 4.88 Mev doublet, the ^209^Po lower energy α at 4.622 MeV (0.548 % of the decay) can be clearly seen.

Despite all of the source preparation difficulties, the original Ag-backed sources were adequate for establishing α-emitting impurity limits with high-resolution PIPS detectors (Sec. 2.2.4), particularly for the most common and expected ^208^Po contaminant. No α-emitting impurities were observed. Figure 13 shows a representative spectrum obtained after about 4 days of counting. From this and other similar spectra, impurity limits for any α emission rate in comparison to the ^209^Po SRM 4326a *E*_α_ are < 10^−3^ for α energies ≤ 4.5 MeV and 2▪10^−5^ for α energies ≥ 5.0 MeV. More specifically, examination of spectra for the ^208^Po α line at 5.122 MeV could be used to establish a ^208^Po/^209^Po activity ratio of < 10^−7^. Despite serious tailing on the lower-energy shoulder of the ^209^Po 4.88 MeV doublet, the 0.548 % α-transition peak at 4.622 MeV is readily observable (Fig. 13).

The serious energy-loss tailing in these Ag sources as well as those in the Pt-backed, mixed ^209^Po + ^241^Am sources precluded any quantitative assays to confirm the LS-based *E*_α_ for SRM 4326a.

### 4.5 Certification of SRM 4326a and Uncertainty Assessment

The prepared and standardized ultrapure, carrier-free ^209^Po solution standards were certified and will be disseminated as NIST SRM 4326a with the following specifications:

**Table:** 

Radionuclide:	polonium-209
Reference Time	1200 EST, 01 December 2013
Massic alpha-particle emission rate of the solution	39.01 s^−1^•g^−1^
Relative expanded (*k*=2) uncertainty	0.46 %

The solution was also characterized in terms of the following uncertified information:

**Table:** 

Source description	Liquid in a flame-sealed 5 mL, Borosilicate-glass ampoule
Solution composition	2.0 mol•L^−1^ HCl
Solution density	(1.032 ± 0.002) g•mL^−1^ at 20 °C
Solution mass	(5.169 ± 0.003) g
Impurities	None detected (see Sec. 4.4)
^209^Po half-life	(125.2 ± 3.3) a

The uncertainty assessment for the ^209^Po massic alpha-particle emission rate for SRM 4326a is summarized in Table 6.

## 5. Summary

The standardization for NIST SRM 4326a, in terms of the massic alpha-particle emission rate for an ultra-pure, carrier-free ^209^Po solution, was based on 4πα LS spectrometry using a unique spectral analysis procedure. It was designed and developed specifically for the case of ^209^Po, accounting for the ^205^Pb 2-keV delayed isomeric transition and for the radiations arising from the 0.45 % EC branch to ^209^Bi.

The standard, contained within a 5 mL borosilicate glass, flame-sealed ampoule, consists of (5.169 ± 0.003) g of a nominal 2.0 mol▪L^−1^ HCl solution (with density of (1.032 ± 0.002) g▪mL^−1^ at 20 °C) and is certified to have a (39.01 ± 0.18) s^−1^▪ g^−1^ massic α emission rate at a reference time of 1200 EST, 01 December 2013. No radionuclidic impurities are known. Uncertainty intervals cited here are for coverage factors of *k* = 2 on the standard uncertainty.

The standardization made relatively exhaustive efforts to test for and ensure solution stability. This investigation included sampling from 13 different gravimetrically linked solutions at various times and conditions. Three LS sources (with one exception of a heavily quenched series of six) were prepared from each solution. Variations included use of two different scintillation fluids, differences in aqueous fractions and acidity of the cocktails, and imposed chemical quenching. There were 42 separate LS sources in all. Each source was measured three times (typically) in two different LS spectrometers on either one or two separate measurement occasions. There were 297 separate LS determinations in all. The measurements were performed over a period of 276 days: 25 days in March 2013; and 31 days in November to December 2013. The certified value was derived from 31 mean values of grouped results. Typical precision (relative combined standard deviation of the mean that included both within-source and between-source variability) on any mean value was 0.14 %. The relative standard deviation on all 31 mean values was 0.17 %.

The work represented in this paper is the latest chapter of many in the past 25 years that involves our laboratory in seminal research on ^209^Po decay and the development of standardization methods and standards for ^209^Po.

## Figures and Tables

**Table 1:** Chronological summary of the NIST/NBS activities involving polonium isotopes ^210^Po, ^208^Po, and ^209^Po. Refer to text (Sec. 1) for details.

Date	Activity	Principal Investigators	Reference
c. 1950s	^210^Po calibrations	H.H. Seliger, W.B. Mann, et al.	[18]
1968	backscattering studies with ^210^Po	Hutchinson et al.	[19]
1984	^208^Po SRM 4327	J.R. Noyce	[20]
1990	Po solution stability	R. Collé	[7]
1993	^209^Po SRM 4326	R. Collé, Z. Lin, et al.	[6,8]
1994	^209^Po decay scheme studies	F.J. Schima, R. Collé	[10]
1994	^205^Pb isomeric state/LS implications	R. Collé, Z. Lin, et al.	[9]
2005	^209^Po SRM 4326 re-certification	R. Collé, L. Laureano-Perez	[8]
2005	^209^Po half-life discrepancy	R. Collé, L. Laureano-Perez, I. Outola	[11]
2006	^210^Pb SRM 4337	L. Laureano-Perez, R. Collé, R. Fitzgerald	[14,15]
2007	^209^Po & ^210^Pb links	R. Collé, L. Laureano-Perez, I. Outola	[11,16]
2008	^210^Pb comparison with NPL	R. Collé, L. Laureano-Perez	[16]
2013	new ^209^Po methodology	R. Collé, R. Fitzgerald, L. Laureano-Perez	this work
2013	^209^Po SRM 4326a	R. Collé, L. Laureano-Perez	this work, ^[17]^
2014	^209^Po definitive half-life	R. Collé, R. Fitzgerald, L. Laureano-Perez	[13]

**Table 2:** Summary of the 13 sample series for the linked ^209^Po solutions as delineated in the scheme of Fig. 1. Each series is described in terms of the solution origin in the scheme; the 2013 preparation date for the counting sources; the commercial scintillation fluid used to prepare the counting source cocktails (where RS refers to “Ready Safe” and UG to “UltimaGold AB”); the range of solution aliquants used for the sources; the cocktails aqueous fraction and comments on the imposed quenching.

Series	Origin	2013 date	Scintillant	Aliquant (mg)	Aqueous fraction	Comment
X	solution X	3/4	RS	0.03 – 0.06	0.08 – 0.09	
L	solution M	3/7	RS	0.04 – 0.06	0.08 – 0.09	
M2	master ampoule M2	3/12	RS	0.04 – 0.06	0.08 – 0.09	
M4	master ampoule M4	3/12	RS	0.04 – 0.06	0.08 – 0.09	
M3	master ampoule M3	11/1	UG	0.04 – 0.05	0.05 – 0.06	quench varied (*n*=6)
M3a	remainder ampoule M3	11/20	UG	0.04 – 0.05	0.06 – 0.1	small quench
M3b	remainder ampoule M3	11/20	UG	0.04 – 0.05	0.06 – 0.11	large quench
S178	SRM ampoule # 178	11/20	UG	0.2 – 1.	0.06 – 0.09	
S17a	SRM ampoule # 17	11/20	UG	0.2 – 0.9	0.06 – 0.09	small quench
S17b	SRM ampoule # 17	11/20	UG	0.2 – 0.9	0.05 – 0.07	large quench
S89	SRM ampoule # 89	11/20	UG	0.2 – 1.1	0.06 – 0.11	
S89a	remainder ampoule # 89	12/4	UG	0.5 – 0.7	0.05	small quench
S89b	remainder ampoule # 89	12/4	UG	0.5 – 0.7	0.09	large quench

**Table 3:** Summary of all of the radiations from decay of ^209^Po to both ^205^Pb (α decay) and ^209^Bi (EC decay), using data from Ref [26]. The ^209^Po decay scheme is shown in Fig. 4.

Transition	Energy (keV)	Probability per decay (%)	Origin
			
Alpha	4622 (7)	0.548 (7)	α 0-2
	4883 (32)	79.2 (32)	α 0-1
	4885 (32)	19.8 (32)	α 0-0
			
Electron, Auger	5.4 –16.3	0.224 (3)	L (Bi)
	57.5 – 63.4		KLL
	70.0 – 77.1	0.012 (2)	KLX
	82.5 – 90.5		KXY
			
	5.3 – 15.8	0.104 (2)	L (Pb)
	56.0 – 61.7		KLL
	68.2 – 75.0	0.006 (1)	KXY
	80.3 – 88.0		
			
Electron, conversion	172.5	0.1288 (3)	K (Pb) γ 2-1 K (Pb)
	174.8	0.043	K (Pb) γ 2-0 K (Pb)
			
X-ray	9.4 – 15.7	0.141 (2)	L (Bi)
	74.8 – 90.4	0.317 (6)	K (Bi)
	9.2 – 15.2	0.063 (1)	L (Pb)
	72.8 – 87.9	0.164 (7)	K (Pb)
			
Gamma-ray	260.5	0.254 (3)	γ 2-1 (Pb)
	262.8	0.085 (2)	γ 2-0 (Pb)
	896.28	0.445 (7)	γ 1-0 (Bi)

**Table 4:** The decay corrected (125.2 a half-life) ^209^Po massic alpha emission rate *E*_α_ for solution M (gravimetrically linked to that for SRM 4326a) for *n* = 31 mean determinations at Reference Time of 1200 EST, 01 December 2013. Each value is given as a function of the measured series, the LS counter used, and measurement time *T*. Refer to Figs. 1 and 2 and the text (Sec. 2.3.1) for abbreviated designations of series and counters. The quantities *T* and *∆ T* correspond respectively to the measurement midpoint time (as 2013 Julian day number) and to half the time interval for the total measurement time (in days) for that determination. The precision estimator *s*, as a combined standard deviation of the mean, in percent, includes the variance components for within-source variability for 3 replicates (typically) and the between-source variability for 3 sources (typically).

Series	Counter	*T*	*∆ T* (day)	*E* α	*s* (%)
X	B	63.497	0.125	4299.5	0.09
X	W	66.545	0.163	4294.8	0.21
L	B	72.061	0.362	4288.5	0.09
M2	B	72.061	0.362	4291.8	0.14
M4	B	72.061	0.362	4297.4	0.12
X	B	84.857	0.532	4300.3	0.12
L	B	84.857	0.532	4286.3	0.26
M2	B	84.857	0.532	4296.9	0.24
M4	B	84.857	0.532	4288.2	0.33
X	W	89.275	0.693	4295.8	0.09
L	W	89.275	0.693	4284.6	0.11
M2	W	89.275	0.693	4289.2	0.14
M4	W	89.275	0.693	4284.2	0.22
M3	W	308.848	0.225	4294.0	0.20
M3	B	311.682	0.227	4292.3	0.21
S17a	B	326.416	1.911	4279.4	0.45
S17b	B	326.416	1.911	4281.7	0.39
S89	B	326.416	1.911	4285.7	0.31
S178	B	326.416	1.911	4292.7	0.32
M3a	W	324.836	0.259	4296.4	0.29
M3b	W	324.836	0.259	4295.6	0.24
M3a	B	331.468	0.341	4293.8	0.21
M3b	B	331.468	0.341	4280.2	0.35
S17a	W	333.361	1.894	4284.4	0.76
S17b	W	333.361	1.894	4303.1	0.39
S89	W	333.361	1.894	4301.9	0.24
S178	W	333.361	1.894	4306.9	0.30
S89a	W	340.217	1.743	4305.0	0.09
S89b	W	340.217	1.743	4297.7	0.10
S89a	B	345.047	1.328	4286.4	0.28
S89b	B	345.047	1.328	4287.8	0.39
	**mean**			4292.3	
	**between**		*s* (%)	0.17	
	**within (median)**		*s_m_* (%)		0.14
	**within (mean)**		*s_m_* (%)		0.14
	***n***			31	

**Table 5:** Comparison of ^209^Po massic alpha emission rates *E*_α_ for unaggregated subgroups of various measurement variables, each under two contrary conditions. The precision estimator *s* is defined in Table 4 and text (Sec. 4.2). The quantities *n* and *∆* refer to the number of values in the subgroup and the relative difference between the two conditions for the variable, respectively.

Measurement variable	Condition	*E*_α_ (s^−1^ g^−1^)	*s* (%)	*n*	*∆* (%)
2013 time	March	4292.1	0.13	13	+ 0.009
	Nov. – Dec.	4292.5	0.20	18	
counter	Wallac	4295.3	0.18	14	− 0.12
	Beckman	4289.9	0.15	17	
solution (activity level)	Master (high)	4292.1	0.13	19	+ 0.015
	SRM (low)	4292.7	0.23	12	
quench (paired sets)	low	4290.9	0.22	6	+ 0.003
	high	4291.0	0.22	6	

**Table 6:** Uncertainty assessment for the ^209^Po massic alpha-particle emission rate for SRM 4326a, where *u* is a relative standard uncertainty for either a type-A (evaluation by statistical methods) or type-B (evaluation by other methods) assessment [22, 23].

	Uncertainty component	Assessment type	*u* (%)
1	LS measurement precision; standard deviation for *n* = 31 mean determinations, includes both within and between variability for (i) *n* = 3 measurements on each LS counting source; (ii) use of *n* = 2 different LS counters on either one or two separate measurement occasions; (iii) *n* = 3 to 6 sources for each solution/LS cocktail composition; (iv) *n* = 13 separate sets of sources/solutions of varying compositions.	A	0.17
2	Background LS measurement variability and cocktail stability; wholly embodied in component 1.	B	—
3	LS Spectra analysis method, including EC correction (see Appendix); partially embodied within component 1. Refer to Sec. 3 and 4.1.	B	
4	LS detection inefficiency, includes wall effect (see Ref. [32]); partially embodied within component 1.	B	0.01
5	LS response correction for EC branch decay. Refer to Sec. 6, Appendix.	B	0.015
6	Gravimetric (mass) dilution factor. Refer to Sec. 2.2.1.	B	0.025
7	Counting source aliquot mass determinations, includes mass measurement precision^;^ partially embodied within component 1.	B	0.05
8	Decay corrections for ^209^Po half-life uncertainty of 2.6 %.	B	0.011
9	Potential alpha and photon emitting impurities. Refer to Sec. 4.4.	B	0.005
**Relative combined standard uncertainty**			**0.23**
**Relative expanded uncertainty (*k* = 2)**			**0.46**

**Fig. A1:**
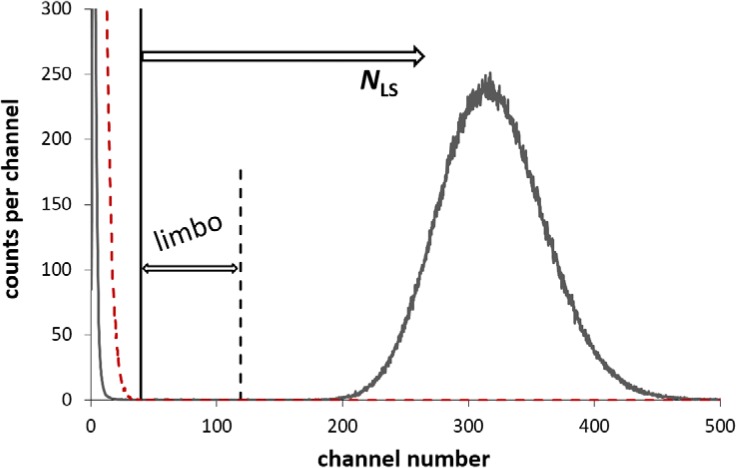
Monte Carlo simulation of LS spectrum for ^209^Po decay, on linear scale. The lower threshold was set using the endpoint from ^3^H (red dashed line).

**Fig. A2:**
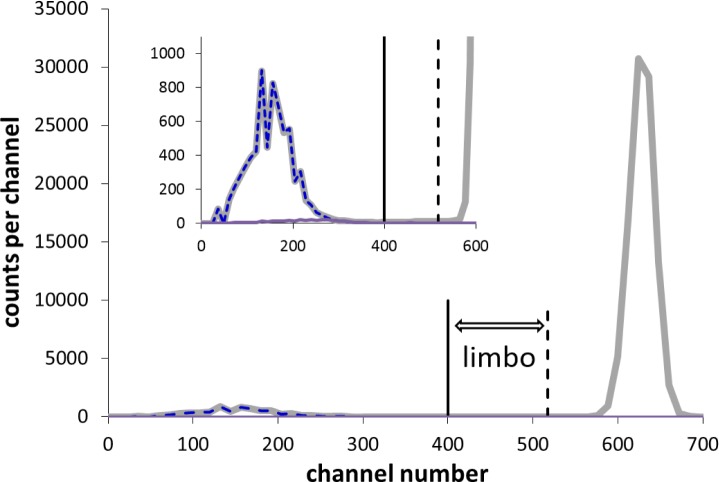
Monte Carlo simulation of ^209^Po LS spectrum, with logarithmic scale. The dashed blue line is the contribution from the 2.3 keV delayed state, assuming a 50 µs extending dead-time in the LS counting system. The low, purple line is the contribution from the EC branch.

## Footnotes

1 Certain commercial equipment, instruments, or materials are identified in this paper to foster understanding. Such identification does not imply recommendation by the National Institute of Standards and Technology, nor does it imply that the materials or equipment identified are necessarily the best available for the purpose.

2 The stated uncertainty is two times the standard uncertainty. Unless otherwise noted all uncertainties given in this paper correspond to standard uncertainties multiplied by a coverage factor of *k* = 2. The treatment and reporting of uncertainties follows the international conventions given in Taylor and Kuyatt [22] and JCGM [23].

## 6. Appendix: Correction for LS Response for Electron Capture Decay to ^209^Bi

In the 1993 standardization of SRM 4326, Collé et al. [6] made a −0.12 % correction to the LS response for the radiations arising from the ^209^Po EC branch. Although the present spectral analysis removed most of the dependence, the massic LS counting rate could still include a small fraction of the EC branch radiations.

Another aspect of the present spectral analysis is that some small fraction of the total alpha events could be lost below the LS counting threshold, set at the apparent ^3^H endpoint in the LS spectra.

Both of these effects have been combined in a correction factor, *k*, that relates the true number of alpha emissions, *N_α_*, to the integral counts above threshold, *N*_LS_, for a given LS measurement.

$$N_{\alpha }=kN_{\text{LS}}.$$ (A1)

Both the loss of alpha response and gain of that from EC decay were estimated in the present work using a Monte Carlo simulation of the LS counting experiments. The Monte Carlo had been built with GEANT4 software [33] that had recently undergone considerable development and testing for simulating the NIST LS-based beta-gamma coincidence counter. The total alpha probability was taken to be *P_α_* = 1 − *P*_EC_ = 0.99546 ± 0.00007 [26]. The simulation was run separately for tritium and ^209^Po, with the overlay of the spectra (Fig. A1) used to set the thresholds, mimicking the real experiment. The simulation was run for 10^6^ alpha decays, and 10^6^ EC decays. The results were scaled by the appropriate branching ratios and then combined to simulate the ^209^Po spectrum.

The correction factor was then determined from the Monte Carlo results, which were analyzed in the same way as the experimental data.

$$k={\left(\frac{N_{\alpha }}{N_{\text{LS}}}\right)}_{\text{MC}}=\frac{995,460}{995,644}=0.99981.$$ (A2)

That is, the correction amounts to a −0.019 % change in the measured LS count rate.

In order to appreciate the nature of this correction, it can be written in terms of the alpha inefficiency correction, *c_α_*, and the correction for the undesired EC contribution, *c*_EC_.

$$k\approx 1+c_{\alpha }+c_{\text{EC}}$$ (A3)

$$c_{\alpha }=1-\varepsilon_{\alpha }$$ (A4)

$$c_{\text{EC}}=-\frac{P_{\text{EC}}\;\varepsilon_{\text{EC}}}{P_{\alpha }\;\varepsilon_{\alpha }}.$$ (A5)

The log-scale spectra in Fig. A2 illustrate these spectral components, as well as events resulting from the 2.3 keV delayed state, which do not contribute to the correction since they fall below the ^3^H threshold. The respective contributions to *k* are *c*_α_ = 0.031 % and *c*_EC_ = −0.050 %.

The uncertainty on *k* included 0.02 % from stochastic variability in the Monte Carlo simulation and 0.010 % from decay data. The standard uncertainty in matching the model to the data was judged by adjusting the threshold by ± 10 channels (linear scale), which changed *k* by 0.011 %.

The value and uncertainty for the spectral correction is therefore, *k* = 0.99981 ± 0.00025

Probably the most challenging aspect of the model was the low-energy scattering of alphas, presumably due to *μ*m-scale boundary (wall) effects. To assess the veracity of the Monte Carlo model for this effect, the fraction of simulated alpha counts landing in limbo in the model (0.08 %) was compared to an experimental value of (0.11 ± 0.02) %, with values ranging from 0.05 % to 0.17 %. This result was obtained from 13 carefully examined spectra obtained with the Beckman counter. The uncertainty (0.02 %) is the standard deviation for the 13 evaluated sources. The reasonable agreement between the model and experiment for the fraction of counts in limbo imbues confidence in the model calculation of the smaller fraction of alpha counts below the ^3^H threshold.

Had the overall correction been more significant, then additional work on the model, and measurements using a pure alpha emitter, would have been warranted. Yet for the present experiment, which was designed to mitigate the effects of *c_α_* and *c*_EC_, the current model is perfectly adequate.

It may be of interest to comment on the correction applied previously by Collé et al. [6, 11, 13] for SRM 4326 and as used for the ^209^Po half-life determination. That previous work used a correction of *k* = −0.12 %, with an estimated uncertainty of about ± 0.05 %. On invoking the same Monte Carlo simulation as described above to the previous spectral analysis approach, the calculated result is −0.08 %, with *c*_EC_ = −0.09 % and *c_α_* = + 0.01 %, which is in reasonable agreement. Further reflection on the original analysis in the Appendix of Ref. [6] suggests that the absolute value of the correction was overestimated based on the assumed LS efficiency for L-capture events. It is also critical to realize that the half-life determination was not affected by the value of the correction since the identical value was used for all decay data during the five measurement periods in 1993, 1994, 2005, and 2013.
